# Posterior mediastinal extramedullary hematopoiesis secondary to hypoxia

**Published:** 2016-05-16

**Authors:** A Solazzo, V D’Auria, LG Moccia, A Vatrella, M Bocchino, G Rea

**Affiliations:** 1. Department of Diagnostic Imaging - Istituto Nazionale Tumori Fondazione G. Pascale - IRCCS, Naples, Italy; 2. Department of Radiology, Fondazione Poliambulanza Istituto Ospedaliero, Brescia, Italy; 3. Respiratory Medicine Division, Department of Clinical Medicine and Surgery, Federico II University, Naples, Italy; 4. Department of Medicine and Surgery, University of Salerno, Salerno, Italy; 5. Department of Diagnostic Imaging, Monaldi Hospital, Naples, Italy

**Keywords:** extramedullary hematopoiesis, posterior mediastinal masses, hypoxia

## Abstract

Two mediastinal masses were incidentally detected at high resolution computed tomography (HRCT) of a 72 year-old male patient, former smoker, affected by chronic obstructive pulmonary disease with worsening dyspnea and 2-year medical history of polycythemia secondary to hypoxia. Integration with a multidetector computed tomography (MDCT) scan after administration of intravenous injection contrast medium showed slightly inhomogeneous increase of enhancement of masses, suggesting in the first case potential malignancy. Diagnosis of extramedullary hematopoiesis was achieved by fine needle aspiration citology (FNAC). Extramedullary hematopoiesis must be considered in differential diagnosis in patients with medical history of polycythemia and severe hypoxia.

## CASE REPORT

A 72 year-old male patient was admitted to the department of Respiratory Medicine Division with a 10-day history of worsening dyspnea also with minimal exertion and mucopurulent sputum to chronic obstructive pulmonary disease (COPD). He presented a 2-years medical history of polycythemia secondary to hypoxia, and for these reasons underwent periodic phlebotomies. He was smoker (30 cigarettes / day for 45 years) with a history of pulmonary heart disease with moderate pulmonary hypertension, diabetes mellitus, hypertension and respiratory failure, treated with oral hypoglycemic agents, diuretics, acetylsalicylic acid, and O_2_ therapy for 24h/die.

Body temperature was normal. Physical examination showed dyspnea at rest, acral cyanosis and peripheral edema and splenomegaly. Physical examination of the chest revealed bronchostenosis and rales on both lower field of lungs.

Laboratory analysis showed the following: red blood cell (RBC) 6.41×10^12^/l; hemoglobin (Hb) 15 g/dl; mean corpuscular volume (MCV) 86.3 fl; hematocrit (HCT) 55.3 %; mean corpuscular hemoglobin (MCH) 23.4 pg; mean corpuscular hemoglobin concentration (MCHC) 27.1 g/dl; red blood cell distribution width - coefficient variation (RDW-CV), 19.1%; red blood cell distribution width - standard deviation (RDW-SD) 56.1 fl; WBC 8.18×10^9^/l; PLT 127×10^9^/l

Arterial blood gas analysis showed an hypoxaemic hypercapnic respiratory failure, with respiratory acidosis : pH 7.329; pCO_2_ 69.7 mmHg; pO_2_ 42.0 mmHg; cHCO^−^_3_ 29.8 mmol/L; cBase: 9.6 mmol/L; flow-volume spirometry test showed a restrictive pattern with obstructive component : forced vital capacity (FVC) 35% (teor. 3.12 lt, meas. 1.08 lt); forced expiratory volume 1 second (FEV_1_) 31% (teor. 2.39 lt, meas. 0.75 lt) ; Tiffeneau Index FEV1/FVC 74.3%; peak expiratory flow (PEF) 32% (teor. 7 lt/sec, meas 2.21 lt/sec) ; forced expiratory flows FEF_25%_ 19% (teor. 6.29lt/sec, meas. 1.21 lt/sec); FEF_50%_ 15% (teor. 3.56, meas. 0,52 lt/sec); FEF_75%_ 27% (teor. 1.01 lt/sec, meas. 0.27 lt/sec). Coagulation profile, amylase and lipase levels were normal. Hepatitis screen was negative.

**Fig. 1.**
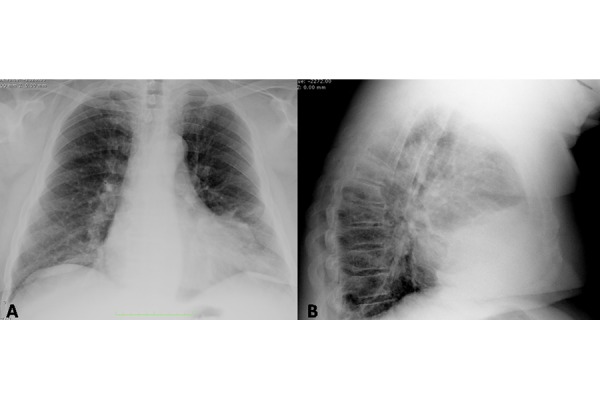
A 72 years-old male affected by polycythemia and COPD. (1A) Postero-anterior and (1B) lateral chest X-Ray show diffuse thickening of peribronchial interstitium. The spine (1B) does not show pathological findings.

Chest X-Ray during his workup in the hospital showed diffuse thickening of peribronchial interstitium; spine did not show any X-Ray abnormality (Fig. 1a–b).

**Fig. 2.**
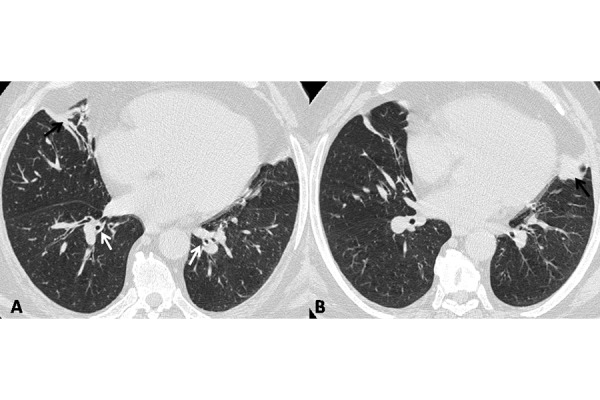
Axial view of HRCT shows the peribronchial thickening (white arrows) and peripheral areas of parenchymal consolidation (black arrows) into the middle lobe and lingula.

Chest high resolution computed tomography (HRCT) showed the peribronchial thickening, typical of COPD and peripheral areas of parenchymal consolidation in the medial segment of the middle lobe and lingula; interstitium was not tickened. (Fig. 2a–b).

**Fig. 3.**
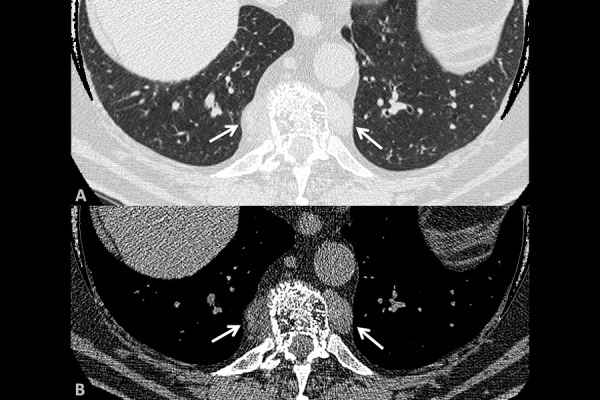
Axial view of HRCT shows two masses (white arrows) occupying left and right paravertebral mediastinal regions.

HRCT scan of the chest showed two masses occupying left and right paravertebral mediastinal regions, that required a volumetric multidetector computed tomography (MDCT) scan before and after intravenous administration of contrast medium (Fig. 3).

**Fig. 4.**
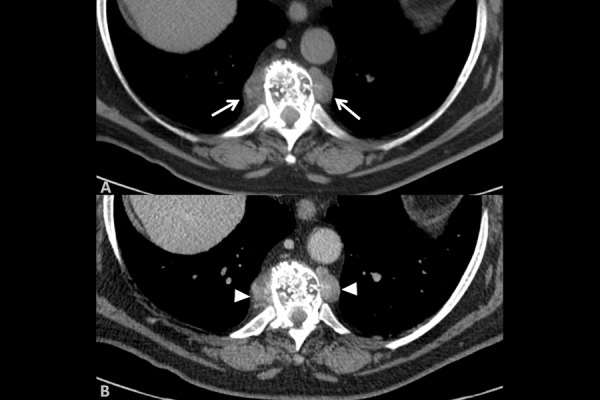
Axial view of MDCT shows: (4A) two lobulated masses (white arrows) with dense soft parts and smooth borders, Masses were located into the posterior mediastinum showing a paravertebral distribution at the level of the tenth thoracic vertebra (4B) After intravenous administration of contrast medium, the masses showed slightly inhomogeneous density enhancement (white arrowhead).

MDCT scan confirmed the presence of two lobulated masses with dense soft parts; borders were smooth and there was not calcification inside. Masses were located into the posterior mediastinum showing a paravertebral distribution at the level of the tenth thoracic vertebra (Fig. 4A). After intravenous administration of contrast medium, masses showed slightly inhomogeneous density enhancement (Fig. 4B).

**Fig. 5.**
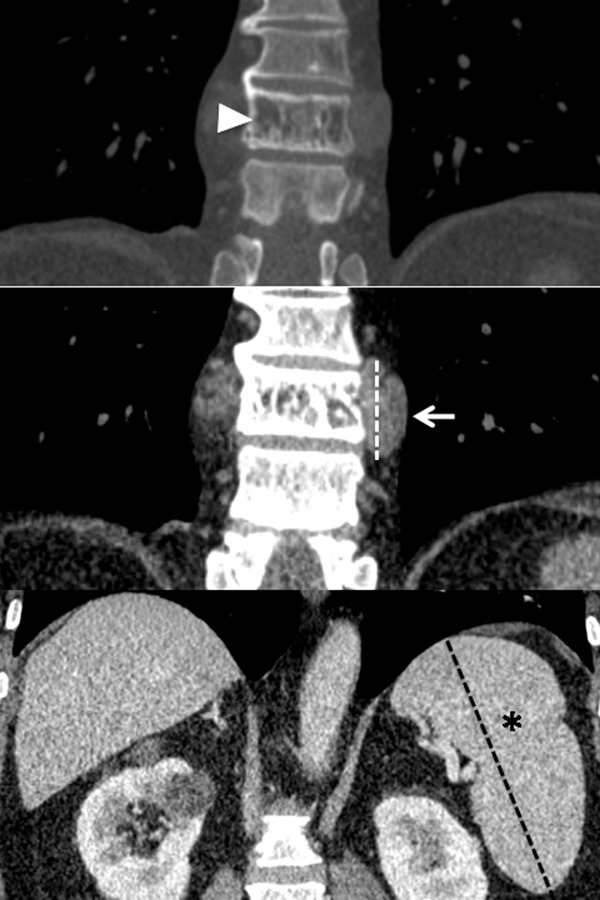
Coronal view of MDCT after intravenous administration of contrast medium: (5A) demineralization of the 10^th^ thoracic vertebra (white arrowhead) without bone erosion; (5B) masses’ craniocaudal extension (white arrow); (5C) splenomegaly (asterisk).

Multiplanar reconstructions (MPR) at the anatomic levels of symmetric lesions were performed. Maximum extension was 33 mm on coronal view. Tenth thoracic vertebra, showed demineralization signs compatible with angiomatous lesion without bone erosion. Other finding was splenomegaly (bipolar diameter 140 mm; normal value 90–120 mm) (Fig. 5a-b-c). Lymphadenopathy and hepatomegaly were not detected Considering the haematological exams, history and risk factors (the patient was a former heavy smoker) with radiological findings and absence of specific signs and symptoms other than a COPD reactivation we considered the differential diagnosis with neurogenic tumors.

**Fig. 6.**
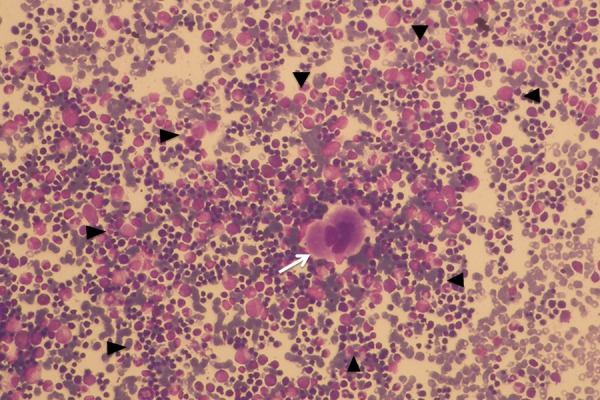
Fine-needle aspiration cytology of right paravertebral mediastinal mass at the level of the 10^th^ thoracic vertebra shows trilineage hematopoiesis with complete cellular maturation characteristic of active hematopoietic tissue. Megakaryocyte in the middle (white arrow) of the field and small clusters of erythroblasts (black arrowhead) without evidence of adipocytes are typical findings for active hematopoietic tissue.

CT guided percutaneous fine needle aspiration cytology (FNAC) was performed on left paravertebral mass and confirmed the diagnosis of extramedullary hematopoiesis (EMH) (Fig. 6).

The patient started a closer follow up for respiratory failure and hematological changes; with a more adequate therapy with maximum dose of bronchodilator drugs (beta2 agonists with corticosteroids 50/500 mcg twice a day and anticholinergic drugs once a day) and an increase of O2 to 2 lit. per minute at rest and during night, and 2.5 lit. per minute under exercise during 24 hours, with bi-level positive airway pressure (BPAP), the last hematocrit (HTC) was 50.6 %, the red blood cells count (RBC) was 5.85×10^12^/lit, hemoglobin (Hb) was 13.7 g/lit, while the CT scan confirmed the approximately same dimensions of masses. The patient underwent to a single phlebotomy during follow up.

## IV. DISCUSSION

EMH is considered a physiological compensatory mechanism, characterized by proliferation of hematopoietic cells outside the bone marrow, occurring when the bone marrow is unable to satisfy the physical demand [1].

EMH is often associated with congenital hemoglobinopathies (thalassemia, sickle cell anemia, and spherocytosis) or with acquired bone marrow replacement disorders (leukemia, lymphoma, myelodysplasia, and myelofibrosis) [2–5].

Extramedullary hematopoiesis tissue usually develops in sites related to hematopoiesis during fetal development [6–8]; experimental studies suggest that muscular and neural tissues can produce hematopoietic cells when stimulated by certain conditions; this effect is given by subpopulations of cells that possess pluripotent potential as reminiscence of embryonic stem cells, and remained unrestricted in specific tissues [9]; the most common sites include liver, spleen and lymph nodes. It may occurs also in kidneys, pleura, skin, ovaries, intestine, sclera, central nervous system, epidural space, and adrenal glands, less common is intra-thoracic EMH [10–11]. EMH causes usually no symptoms and develops slowly over time, but spinal cord compression [6] and spontaneous rupture [12] have been described. The most common presenting symptom into malignant neoplasms is local vertebral pain that may be accompanied by radicular pain and paresthesia.

EMH causes usually no symptoms and findings are incidental. Diagnosis of intra-thoracic EMH can be suspected on a chest X-Ray, CT or magnetic resonance imaging (MRI) [13].

Chest X-Ray is often normal, but if intra-thoracic EMH is large, it may show paravertebral lobulated masses with well-defined borders, usually bilateral, without calcifications or bone erosion. Bone lesions, if present, are related to underlying disease [14–16].

On CT scan, intra-thoracic EMH is characterized by presence of lobulated masses with dense soft parts, usually homogeneous with smooth borders, without calcification inside and may be present adipose tissue within the mass. Usually those masses are bilateral and located below the level of the sixth thoracic vertebra [17–18]. After intravenous administration of contrast medium, the masses show homogeneous density enhancement because of their high vascularity [19], but CT scan may reveal an inhomogeneous density enhancement if occurs fat infiltration.

MRI may be useful to identify adipose tissue within the mass and demonstrate thoracic rib expansion without evidence of cortical bone erosion. EMH masses usually present low signal on both T1w and T2w images; after intravenous administration of contrast medium, is evidence of low enhancement, although EMH tissue is highly vascular [19].

Radionuclide bone scintigraphy using ^99^mTc sulfur colloid or ^111^In has a role not yet been clarified, but it can be used to demonstrate areas of focal uptake of the tracer localizing to paravertebral masses (hematopoietic activity) [20].

The diagnosis can be obtained with FNAC, open biopsy, or thoracoscopic biopsy in uncertain cases [21].

Other causes of posterior mediastinal masses include neurogenic tumors (such as schwannoma, neurofibroma and ganglioneuroma), benign bone tumors of the spine (such as aneurysmal bone cyst and fibrous dysplasia), mesenchymal tumor, hemangioma, lymphangioma, malignant neoplasms (metastases, lymphoma, multiple myeloma, and Ewing sarcoma), infective etiologies (tuberculosis, nocardia and actinomyces) [19]. Calcifications in patients with EMH is demonstrated extremely rarely [22]. In tuberculosis, lymph nodes show commonly peripherally enhancement [23]; in lymphomas, enhancement is usually slight and homogeneous. Neurogenic tumors of the posterior mediastinum commonly have erosions or defects of the vertebrae or ribs and may show moderate homogeneous contrast enhancement. EMH is exclusively extradural, a feature that cannot always be distinguished with imaging, while schwannomas occur most commonly in the context of meningeal dural layer, but they can occur outside of meningeal dural layer [24]. EMH treatment includes corticosteroids, blood transfusion, hydroxyurea, splenectomy in cases of spherocytosis, surgical decompression or radiation therapy [21].
